# Prognostic Molecular Signatures for Metastatic Potential in Clinically Low-Risk Stage I and II Clear Cell Renal Cell Carcinomas

**DOI:** 10.3389/fonc.2020.01383

**Published:** 2020-08-11

**Authors:** Andrew J. Shih, Neal Murphy, Zachary Kozel, Paras Shah, Oksana Yaskiv, Houman Khalili, Anthony Liew, Louis Kavoussi, Simon Hall, Manish Vira, Xin-Hua Zhu, Annette T. Lee

**Affiliations:** ^1^Feinstein Institutes for Medical Research, Manhasset, NY, United States; ^2^Donald and Barbara Zucker School of Medicine at Hofstra/Northwell, Hempstead, NY, United States; ^3^Division of Hospital Medicine, LIJ Medical Center, New Hyde Park, NY, United States; ^4^The Smith Institute for Urology, New Hyde Park, NY, United States; ^5^Department of Urology, Mayo Clinic, Rochester, MN, United States; ^6^Northwell Health Department of Pathology, New Hyde Park, NY, United States; ^7^Northwell Health Cancer Institute, Lake Success, NY, United States

**Keywords:** clear cell, molecular biomarker, renal cell carcinoma, gene expression, miRNA, canonical correlation analysis

## Abstract

**Introduction:** For patients with localized node-negative (Stage I and II) clear cell renal cell carcinomas (ccRCC), current clinicopathological staging has limited predictive capability because of their low risk. Analyzing molecular signatures at the time of nephrectomy can aid in understanding future metastatic potential.

**Objective:** Develop a molecular signature that can stratify patients who have clinically low risk ccRCC, but have high risk genetic changes driving an aggressive metastatic phenotype.

**Patients, Materials, and Methods:** Presented is the differential expression of mRNA and miRNA in 44 Stage I and Stage II patients, 21 who developed metastasis within 5 years of nephrectomy, compared to 23 patients who remained disease free for more than 5 years. Extracted RNA from nephrectomy specimens preserved in FFPE blocks was sequenced using RNAseq. MiRNA expression was performed using the TaqMan OpenArray qPCR protocol.

**Results:** One hundred thirty one genes and 2 miRNA were differentially expressed between the two groups. Canonical correlation (CC) analysis was applied and four CCs (CC32, CC20, CC9, and CC7) have an AUC > 0.65 in our dataset with similar predictive power in the TCGA-KIRC dataset. Gene set enrichment showed CC9 as kidney development/adhesion, CC20 as oxidative phosphorylation pathway, CC32 as RNA binding/spindle and CC7 as immune response. In a multivariate Cox model, the four CCs were able to identify high/low risk groups for metastases in the TCGA-KIRC (*p* < 0.05) with odds ratios of CC32 = 5.7, CC20 = 4.4, CC9 = 3.6, and CC7 = 2.7.

**Conclusion:** These results identify molecular signatures for more aggressive tumors in clinically low risk ccRCC patients who have a higher potential of metastasis than would be expected.

## Introduction

In 2020, the American Cancer Society estimates 74,000 new cases of renal cell carcinoma will be diagnosed and account for ~15,000 deaths, putting renal cell carcinoma in the top ten leading cause of cancer deaths in the United States (1). Of the three major subtypes (clear cell, papillary, and chromophobe) clear cell renal cell carcinoma (ccRCC) is the most prevalent comprising 75% of diagnosed cases and resulting in more deaths per year than other subtypes (1).

For most ccRCC patients with early stage localized tumor, stage I or II, surgical resection offers achievable survival rates over 95% (1). However, for a small percentage of clinically low risk patients disease recurrence ensues in 5–10% stage I and 15–20% stage II patients. Several models exist to assess risk for disease recurrence after nephrectomy including: MSKCC postoperative prognostic nomogram (2), UCLA UISS score (3) and Mayo clinic SSIGN score (4). These scoring systems rely on clinical parameters such as tumor grade, size, presence of necrosis, and patient functional status that often do not accurately differentiate early stage clinically low risk disease. Only tumor size has the highest prognostic accuracy for future development of metastatic disease (5).

While new treatments have increased survival, <20% of patients diagnosed with metastatic disease have a median survival of >27 months (6). Kuijpers et al. estimated that 71% of stage I and 52% of stage II RCC patients who developed metastatic disease following surgery were potentially curable, reasoning that early detection of metastatic disease would lead to more successful treatment (7). However, Dabestani et al. reported that intensive follow-up after nephrectomy does not improve overall survival after recurrence (8).

Therefore, patients with clinicopathologically defined low risk ccRCC (stage I and II) who develop a distant recurrence within 5 years represent a unique cohort of patients, where understanding the biology of the key molecular factors driving progression to metastatic disease can help supplement clinical parameters. Given that there are a relatively small number of people who develop metastasis in early stage ccRCC, we chose to match a similar number to have a balanced dataset that can capture as many molecular signals as possible. Here we present an analysis of a similar number of tumor samples from stage I and II ccRCC patients who developed metastatic disease within 5 years of surgery and from patients who did not develop metastatic disease in more than 5 years to capture the molecular signatures that associate with metastatic potential at an early time point as possible. Furthermore, we validated these signatures in The Cancer Genome Atlas ccRCC (TCGA-KIRC) cohort.

## Patients, Materials, and Methods

### Patient Selection

Patients from 2008-2012 who had metachronous metastases within 5 years with a primary diagnosis of stage I or II ccRCC in the Northwell Health tumor bank were selected. A similar number of patients were selected who did not have recurrence as a comparison group to maximize the molecular signatures captured. The ccRCC tissue samples in the discovery set were obtained from the Northwell Health pathology department as formalin-fixed, paraffin-embedded (FFPE) tissue blocks. Samples were initially matched on size, gender and age with two samples removed for QC issues following RNA extraction.

TNM classification and the Fuhrman grading system were used to stage and grade tumor samples (9, 10). The Northwell Health System Regional Ethics Committee granted research approval for the study with waivers of HIPAA Authorization and informed consent. The validation set consisted of 218 Stage I and 45 Stage II patients from the TCGA-KIRC Data Resource (11).

### RNA Extraction

A genitourinary pathologist (O. Yaskiv) reviewed FFPE tissue blocks and their corresponding H&E slides. Slides were matched up with tissue blocks and ~35 mg of the pre-identified tumor were excised. RNA was extracted using the RecoverAll^TM^ Total Nucleic Acid Isolation Kit according to manufacturer's instructions. RNA was evaluated using an AB Bioanalyzer.

### mRNA Sequencing and Expression Analysis

Libraries were prepared using the Illumina TruSeq RNA Access Library kit and sequenced on a NextSeq 500 following the manufacturer's instructions. Sequenced segments were aligned with STAR2 (12) to the Human GENCODE reference (13). Gene counts were assessed using ht-seq counts (14). Differential expression was calculated using DESeq2 which is designed to analyze digital count data generated by sequencing (15). Log fold changes were shrunk using apeglm (16). Pathway enrichment analysis was done using GAGE (17).

### Cell Subset Deconvolution

Composition of cell subsets of our bulk RNA-seq ccRCC was determined with CIBERSORT (18) using a previously published ccRCC single cell RNA-seq dataset (19) as a reference. Only cell populations present in >1% of ccRCC samples were considered. Correlations of cell populations to metastatic phenotype was done using MASC (20).

### miRNA RT-qPCR and Expression Analysis

Quantification of miRNA was done using the TaqMan OpenArray, which profiles 754 known miRNAs using qPCR. A Ct threshold of 30 was used for detectable level of expression. MiRNAs were further filtered by removing those with no expression in any samples and samples were filtered that had <10% of miRNAs detected. Housekeeping miRNAs were the top 10 most stable miRNAs from the NormqPCR package (21), which were used to normalize raw Ct values using ΔCt normalization (22). Differential expression was calculated using limma which is designed to analyze log2 based expression assays like qPCR (23).

### Canonical Correlation Analysis and Validation

*De novo* mRNA and miRNA modules or Canonical Components (CCs) were identified in the discovery set using sparse canonical correlation analysis (24) with AUC to metastatic status calculated using pROC (25). Data analysis was done using R (26) and tidyverse (27). RNA-seq and microRNA-seq count data for validation were done in the TCGA-KIRC dataset which was downloaded, then normalized using DESeq2. Phenotypes were defined using the TCGA Pan-Cancer Clinical Data Resource (28). The specific gene and microRNA weights calculated for CCs in the discovery set were applied to the validation set. Thresholds for risk stratification in the discovery set on the discovered CCs were done using rpart (29) and then applied to the validation set to create high and low risk groups. Cox regression of the high and low risk groups in the validation set to their disease free survival time was done using survminer (30).

## Results

### Demographics

The discovery set consisted of 24 patients with Stage I and 20 patients with Stage II ccRCC who underwent either partial or radical nephrectomy. In Stage I, 13 patients had more than 5.5 years of follow-up with no evidence of metastasis while 11 developed metastatic relapse within 5 years; for Stage II, 10 patients did not develop metastases, while 10 patients developed metastatic disease (see Table 1). Two samples did not meet QC metrics for microRNA. The population was 16% female (16.7% in stage I and 15% in stage II). The average age at surgery for the entire group was 61.3 ± 10.3 years with no significant differences between stage I (62.2 ± 8.4 years) and stage II (60.3 ± 12.5 years).

**Table 1:** Demographics of patients in this study.

	**Stage I**		**Stage II**	
	**Metastatic (11)**	**Non-metastatic (13)**	**Metastatic (10)**	**Non-metastatic (10)**
Women	1 (9.1%)	3 (23.1%)	2 (20%)	2 (20%)
Size (cm)	4.7 ± 1.2	4.9 ± 1.1	10.7 ± 3	11 ± 3.2
Low grade (I–II)	9 (81.8%)	11 (84.6%)	4 (40%)	5 (50%)
Age at surgery	63.9 ± 6.9	61.8 ± 9.7	58.8 ± 11.1	64 ± 13.7
MSKCC score	91.5 ± 1.4	91.3 ± 1.4	69.7 ± 10.7	68.5 ± 11.9
UISS score	89.2 ± 4.3	89.5 ± 4	80.4 ± 0	80.4 ± 0
Recurrence time	29.7 ± 20.3	N/A	17.1 ± 19.8	N/A
Radical Nephrectomy^*^	2/9 (22.2%)	3/13 (23.1%)	6/6 (100%)	4/4 (100%)
Margins^*^	Neg (9/9)	Neg (13/13)	Neg (6/6)	Neg (4/4)

* *Nephrectomy and margin status were not available for all patients*.

For stage I patients, metastases were evident at 29.7 ± 20.3 months, and the average tumor size of Stage I was 4.78 ± 1.14 cm with 83.3% low grade (I–II) and 16.7% high grade (III–IV). While patients with stage II disease recurred in 17.1 ± 19.8 months, with an average tumor size of 10.82 ± 2.98 cm with 45% low grade and 55% high grade.

There was no significant difference in age, tumor size, grade, radical vs. partial nephrectomy, margin status, UISS and MSKCC recurrence score in patients who developed metastases compared to those who did not: age at surgery (*p*-value stage I=0.54 and stage II=0.36), tumor size (*p*-value stage I=0.71 and stage II=0.82), tumor grade (*p*-value stage I=0.57 and stage II=0.75), radical vs. partial nephrectomy (*p*-value stage I=1 and stage II=1), margin status (*p*-value stage I=1 and stage II=1), UISS recurrence score (*p*-value stage I=0.86 and stage II=1), or MSKCC recurrence score (*p*-value stage I=0.81 and stage II=0.82).

### RNAseq Gene Expression Analysis of Stage I and II ccRCC Tumors

**Figure 1:**
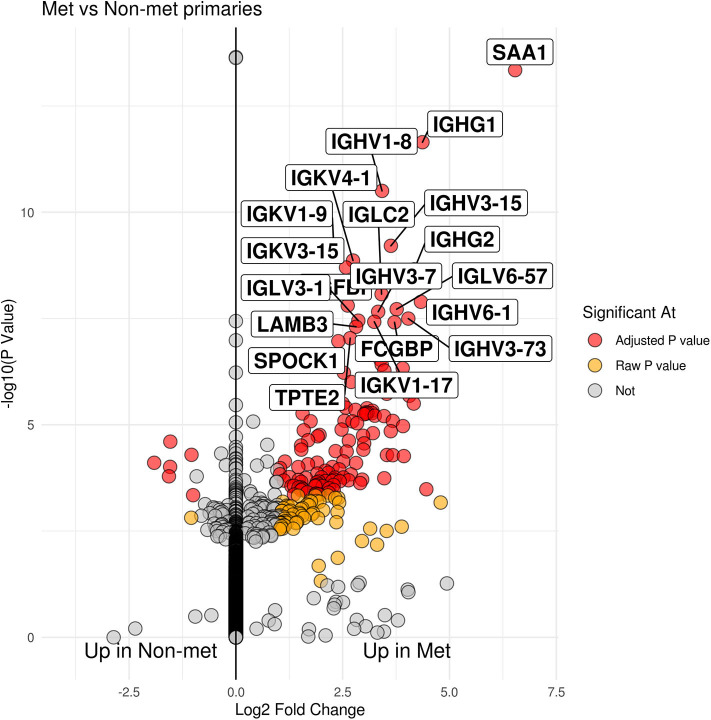
Volcano plot of patients who developed metastases within 5 years vs. those that did not. The x-axis is log 2 fold change between groups while the y-axis is -log 10 raw p-value. The top 20 genes by p-value are labeled.

Group comparison of patients who developed metastatic disease vs. those who did not showed 131 genes that were significant after multiple testing correction (see Figure 1 and Supplementary Table 1). Of those genes, the majority of them were seen in patients that had developed metastases (125 genes) as opposed to patients cured by surgery (6 genes). Seventy two of the 125 genes upregulated in tumors from patients who developed metastatic disease were immunoglobulin genes (IGH, IGK, and IGL).

The model used to develop the molecular signature of metastasis in ccRCC used variables that took into account the contribution of stage and gender to remove those covariates from contributing to the signal for metastases. There were 267 stage specific genes with adjusted *p* < 0.05 with absolute value log 2 fold change > 1; 253 of these were seen up-regulated in stage II while 14 genes were seen to be up-regulated in stage I, see Supplementary Table 2. In gender, 166 genes were found to pass similar thresholds, with 150 genes higher in males with 16 genes higher in females (see Supplementary Table 3). Notably, many of the genes found are gender specific like Y chromosome specific genes (i.e., UTY, DDX3Y, USP9Y, and RPS4Y1) being higher in males or XIST, the X-inactive specific transcript, being higher in females.

**Figure 2:**
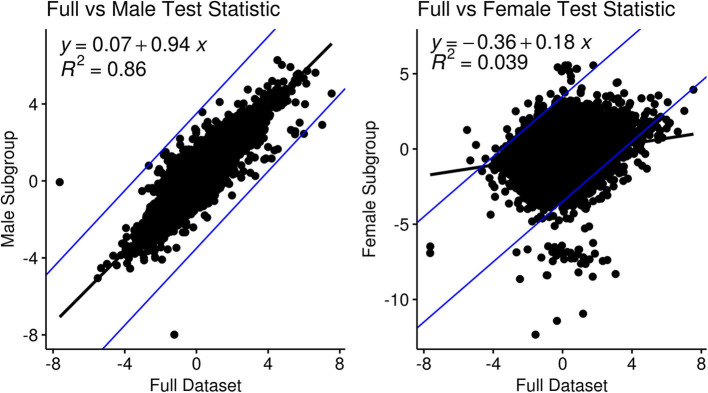
Comparison and correlation of the metastatic test statistic in the full dataset in a sub-group analysis for gender: (left) males and (right) females.

Sub-group analysis focused on male-specific or female-specific signals found a high correlation between test statistics of the male subgroup vs. the full data (y ~ 0.94x, R^2^= 0.86) compared to those of the female sub-group (y~0.18x, R^2^= 0.039), see Figure 2. In the male-specific subgroup analysis 8 genes had an adjusted p-value threshold of 0.05 that was higher than 0.1 in the full group analysis: CNDB2, COL8A2, EPHB2, KRT16, PRSS21, SOGA3, SPINK5, and TMEM63C. In female-specific subgroup analysis, only three genes matched the same criteria, CTHRC1, BCL2L14, and AGBL4.

Deconvolution of the RNA-seq data into cell subsets resulted in 28 distinct cell types (see Supplementary Table 4. None of the cell subpopulations were associated with patients who developed metastases with *p* < 0.05.

### miRNA Expression Analysis of Stage I and II ccRCC Tumors

Since miRNA is much sparser (only 214 miRNA passed QC thresholds, see Supplementary Table 5) the adjusted p-value threshold for miRNA significance was lowered to 0.25. Only two miRNAs were found to be significant at this threshold. MiR-18a and miR-301 were found to be upregulated in patients who developed metastatic disease. Gender and stage were also taken into account in differential expression analysis of miRNA, see Supplementary Tables 6, 7. For gender, there were no miRNA that passed adjusted *p* < 0.25. For stage, 157 total miRNA had adjusted *p* < 0.25 with 121 associated with stage II and 29 associated with stage I.

### Canonical Correlation Analysis With mRNA and miRNA Expression Datasets

Sparse canonical correlation analysis (CCA) is able to identify similar correlation structures between two orthogonal datasets and redefine them as canonical components (CCs). It is analogous to Principal Component Analysis (PCA) with two orthogonal multi-dimensional datasets; making linear combinations of variables that have the highest correlation across datasets. CCs can be thought of as *de novo* modules of genes and miRNAs that have correlated expression patterns with 42 total CCs identified where expression of both can be combined into a single score (see Supplementary Table 8). Univariate logistic regression was performed on canonical components to classify patients who developed metastatic disease. The top performing canonical component (CC32) had an AUC of 0.77 (see Table 2). To assign potential function to CCs, a gene enrichment analysis, GAGE, was used on Gene Ontology (GO) terms with an adjusted p-value threshold of 0.2. CC32 was enriched in GO terms related to RNA binding and spindle function, CC20 was enriched in oxidative-phosphorylation genes, CC9 was enriched in kidney development while CC7 was enriched in immune terms.

**Table 2:** Canonical components (CCs) with AUC > 0.65 in both the discovery and validation datasets.

**Canonical Component**	**Discovery AUC**	**TCGA AUC**	**GO terms**	**Adjusted P-Value**
CC32	0.78	0.74	RNA BINDING	1.36E-01
CC32	0.78	0.74	SPINDLE	1.36E-01
CC20	0.69	0.76	MITOCHONDRION	3.64E-19
CC20	0.69	0.76	MITOCHONDRIAL PART	1.29E-16
CC20	0.69	0.76	CELLULAR RESPIRATION	8.69E-15
CC20	0.69	0.76	SMALL MOLECULE METABOLIC PROCESS	1.44E-14
CC20	0.69	0.76	OXIDATION REDUCTION PROCESS	1.61E-13
CC9	0.68	0.74	MESONEPHROS DEVELOPMENT	1.68E-01
CC9	0.68	0.74	BIOLOGICAL ADHESION	1.68E-01
CC9	0.68	0.74	HOMOPHILIC CELL ADHESION VIA PLASMA MEMBRANE ADHESION MOLECULES	1.68E-01
CC9	0.68	0.74	KIDNEY EPITHELIUM DEVELOPMENT	1.68E-01
CC7	0.65	0.68	REGULATION OF IMMUNE RESPONSE	8.15E-05
CC7	0.65	0.68	EXTERNAL SIDE OF PLASMA MEMBRANE	2.42E-04
CC7	0.65	0.68	REGULATION OF IMMUNE SYSTEM PROCESS	2.42E-04
CC7	0.65	0.68	ADAPTIVE IMMUNE RESPONSE	3.02E-04
CC7	0.65	0.68	IMMUNOGLOBULIN COMPLEX	7.04E-04

### Validation in TCGA-KIRC

A subset of the publically available RNA-seq and microRNA-seq TCGA-KIRC datasets (stage I and II only) was used as validation to evaluate the accuracy of the canonical components identified. After matching RNA-seq and microRNA-seq samples and removing multiple samples from the same patient, the validation set had 263 patients total. Of those, 218 were stage I with 16 of those having recurrences, while of the 45 stage II samples 7 recurred. Gene and microRNA weights for the CC score calculated in the discovery set were applied to the validation set. Both datasets were mean normalized to 0 and standard deviation normalized to 1. The four identified CCs with AUC≥0.65 in the discovery set also had an AUC≥0.65 in TCGA-KIRC.

**Figure 3:**
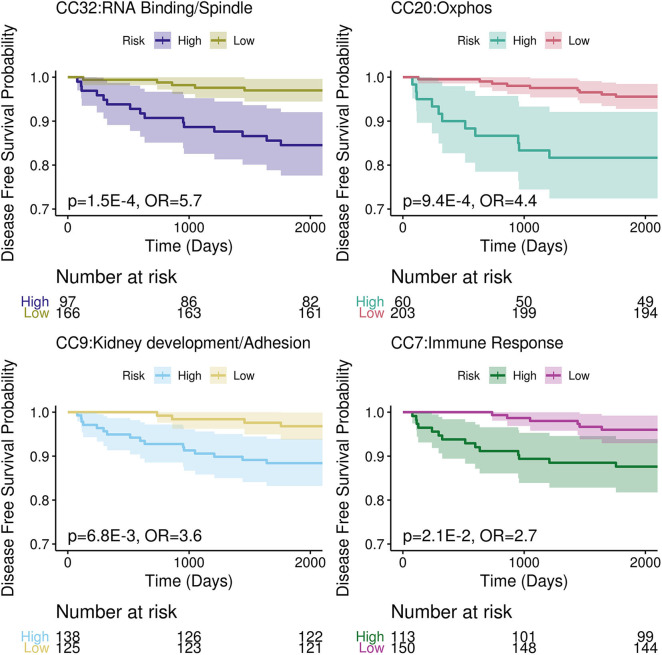
Kaplan-Meier Curves of the high and low risk groups determined by top four CCs. The 95% confidence interval plotted with each group as the lightly shaded area.

To determine accuracy of the CCs, the discovery dataset was used to set thresholds in order to stratify TCGA-KIRC into high risk and low risk groups. Thresholds were set using decision trees in the discovery dataset to maximize separation between groups. These thresholds were applied to TCGA-KIRC to separate them; thresholds for high risk were CC9 <0.02, CC20>0.52, CC32>0.23 and CC7 < -0.04. A cox regression was performed with these high and low risk groups in TCGA-KIRC based on their progression free survival for each CC. The recurrence rate in the validation set was ~9%; assuming an equal split between high and low risk groups (13% recurrence and 5%, respectively), an odds ratio of 2.5 has a power of 0.81. All cox regression analyses were found to be significant (p-values CC9 = 6.8E-3, CC20 = 9.4E-4, CC32 = 1.5E-4, and CC7 = 0.021, see Figure 3) with an odds ratio >2.5 (OR CC32 = 5.7, CC20 = 4.4, CC9 = 3.6, and CC7 = 2.7).

## Discussion

Here we examined tumors from clinically low risk ccRCC for molecular signatures to understand metastatic potential. Risk stratification using clinicopathological parameters in these patients has limited predictive power. In our dataset, we see no statistical significance between MSKCC and UISS scores in patients who do or do not develop metastatic disease in our cohort. Our low risk ccRCC patient cohort who develop metastasis offers an early time point where molecular signatures can supplement clinical information for better risk stratification. To increase molecular signatures found we chose to use similar numbers of patients who did and did not develop future metastatic disease to have a balanced dataset. We report several multigene and miRNA signatures generated by CCA that show clinical utility in stratifying clinically low risk ccRCC tumors.

To the best of our knowledge, this is the first report analyzing the combined differential mRNA and miRNA expression between clinically low risk stage I and II ccRCC with vastly different outcomes. Several recent studies have used gene expression analysis to create stratification scoring systems in RCC: Rini et al. found a 16 gene assay to predict recurrence in stage I-III ccRCC using gene expression across almost 1000 samples (31), Brooks et al. developed ClearCode34 from 72 ccRCC samples (stage I-III) to identify low risk (ccA) and high risk (ccB) groups (32), Morgan et al. examined 31 cell cycle related genes across 565 patients of clear cell, papillary, or chromophobe RCC to create the R-CCP panel (33). However, they included stage III ccRCC patients and did not use miRNA in conjunction with gene expression. In stage III ccRCC, the tumor is no longer completely isolated to the kidney and has already spread either to the most proximal lymph node, to a major vein or to the tissue surrounding the kidney, which indicates a more aggressive phenotype and increased risk. Therefore, the results found in our discovery population and validation in TCGA-KIRC are a novel and a promising step toward identifying molecular signatures of clinicopathologically defined low risk ccRCC that develop future metastasis.

In our analysis, several immunoglobulin genes were upregulated in tumors that became metastatic. IgG is overexpressed in ccRCCs in comparison to adjacent normal tissue affecting cell proliferation, migration and invasion (34). Immunoglobulin genes have been shown to be active and expressed in many non-B epithelial cancer cells (35). MZB1, which is overexpressed in our metastatic group, has been shown to be necessary for immunoglobulin synthesis (36). It also has an immune regulatory effect that has a survival benefit in other cancers (37, 38), though in RCC high expression of MZB1 is unfavorable (11, 39). Similarly IL1R2, which has higher expression in our metastatic samples, is a mock receptor of IL1R that regulates immune response through competitive inhibition, and has an important role in cancer progression (40). Furthermore, our CC7 module is enriched in immune response GO terms. While expression of immune genes is normally associated with immune infiltrating cells, we did not see the future development of metastasis correlate with any specific immune cell type or any cell at all when our bulk RNA-seq data was deconvoluted into cell types via CIBERSORT. These data show that the immune system may play a role in promoting a pro-tumor environment early in patients who develop metastatic disease.

In addition to the immune module (CC7), other *de novo* mRNA-miRNA modules have functions related to cancer growth. CC9 was enriched in GO terms for kidney development and cell adhesion. Enrichment of kidney epithelium terms directly links this module to ccRCC cells, which predominantly are kidney epithelial cells. One of the two miRNAs passed our adjusted p-value threshold, miR-18a, promotes proliferation and inhibits apoptosis in kidney cancer cell lines and is associated with worse overall survival in RCC (41). Furthermore, this particular GO term is related to development from embryos, which implies a more stem-like state compared to a mature kidney, similar to an epithelial-mesenchymal transition. The other microRNA that passed our adjusted p-value threshold, miR-301 which had higher expression in those that developed metastasis, has been shown to be increased in microvesicles released by human renal cancer stem cells to stimulate angiogenesis to prepare the metastatic niche (42). TGFBI is also overexpressed in our metastatic discovery set and has been shown to induce epithelial to mesenchymal transition (43) as well as being associated with ccRCC tumor progression and poor prognosis (44).

One of the hallmarks of ccRCC is the metabolic reprogramming of oxidative-phosphorylation pathways leading to accumulation of lipids and glycogen in the cytoplasm supporting a shift in metabolism known as the Warburg effect (45). Analysis of TCGA-KIRC showed worse survival is associated with upregulation of fatty acid synthesis genes and pentose phosphate pathway genes while better survival was associated with Krebs cycle genes (11). CC20 in our dataset is enriched in cellular respiration and oxidative-phosphorylation terms, and we have shown has predictive value for metastatic potential. One gene related to solute transport that we also see upregulated in the metastatic group is SLC38A5, which alkalinizes tumor cells and promotes growth; it is also a transcriptional target for the oncogene c-Myc (46).

Interestingly, there is a gender disparity in the prevalence of RCC, about 2:1 male to female, that is consistent across age, year, and region (47). Men are more likely to have a higher grade tumor and are more likely to develop metastases, while women showed a benefit in overall survival (48). Some studies have examined mutational differences between men and women in ccRCC, showing that stratification by gender showed BAP1 mutations have a female-specific poorer outcome (49). Similarly, Tan and colleagues also showed expression levels of FABP7 and BRN2 had prognostic value in women (50). Many of the treatment and stratification options bias toward male because of the increased prevalence, which is also present in our dataset (~84% male). We did have three genes that were differentially expressed in women that were not seen in our overall dataset: CTHRC1, BCL2L14 (overexpressed in metastatic patients) and AGBL4 (underexpressed in metastatic patients). CTHRC1 knockdown has been shown to reduce proliferation and epithelial-to-mesenchymal transition (51) and increased expression is associated with a poorer prognosis (11, 39). BCL2L14 is a member of the BCL2 family and AGBL4 is an ATP/GTP binding protein, both without relevance to RCC to the best of our knowledge, but given our results further study may be warranted, particularly in women.

While our study focuses on early stage ccRCC, we have applied previous molecular panels to our dataset with mixed results, likely because of the equal number of patients who would develop metastases vs. those that did not in a low risk cohort (data not shown). We also applied our CCs to stage III patients in TCGA with two of the CCs (CC32: RNA Binding/Spindle and CC20: Oxphos) having the lowest p-value of ~0.08, implying these are worth further study for metastatic progression across all risk groups. One common limitation regarding genomic-based predictive markers is intratumor heterogeneity. However, CCA takes into account similarly correlated genes and miRNA across samples to make *de novo* modules or pathways that are less susceptible to cellular heterogeneity. Furthermore, bulk deconvolution using cell subset techniques did not show any cell type associated with metastases. Using TCGA-KIRC for validation may have been limited due to the fact that the TCGA-KIRC has local and distant recurrence of disease in their dataset when our cohort consisted only of distant metastases. However, 7% of stage I and 15% of stage II in TCGA-KIRC recurred which is similar to the reported distant metastatic rate, making local recurrences less likely.

## Conclusions

Our results highlight molecular signatures that can risk stratify patients for metastatic potential in clinically low risk ccRCC patients. Our modules provide a potential mechanistic pathway for development of metastases, of which the immune module and immunoglobulin genes are of particular interest. With further validation, the combined mRNA and miRNA modules could be used to improve treatment and survival outcomes for this group of patients.

## Data Availability Statement

The datasets generated and analyzed for this study can be found in the Gene Expression Omnibus at identifier GEO: GSE155210.

## Ethics Statement

The studies involving human participants were reviewed and approved by The Northwell Health System Regional Ethics Committee. Written informed consent for participation was not required for this study in accordance with the national legislation and the institutional requirements.

## Author Contributions

ZK, PS, X-HZ, and ATL conceived and designed the study. OY, HK, and AL acquired the data. AS analyzed and interpreted the data. AS, NM, and ATL drafted the manuscript. AS, NM, ZK, LK, SH, MV, X-HZ, and ATL critically revised the manuscript for important intellectual content. AS did the statistical analysis. OY, HK, AL, X-HZ, and ATL provided administrative, technical, and material support. ATL obtained funding and supervised the study. All authors contributed to the article and approved the submitted version.

## Conflict of Interest

The authors declare that the research was conducted in the absence of any commercial or financial relationships that could be construed as a potential conflict of interest.
